# Knowledge, attitudes, and practices toward chemotherapy resistance among oncologists: a multinational cross-sectional study

**DOI:** 10.3389/fonc.2026.1815979

**Published:** 2026-06-05

**Authors:** Bassam Abdul Rasool Hassan, Ali Haider Mohammed, Khalis Mohamed, Vladimir Yu. Startsev, Chema Drira, Omar Hamdy, Miada Mohamed Fahmi Elmetwaly, Said Nabil, Shamma Alshehhi, Gamil Othman, Hamoud H. Y. Al-Hussaini, Ahmed Zuhair Abdulhameed Alsammarraie, Arooj Abid, Nada M. Kassem, Sami El Khatib, Souheil Hallit, Diana Malaeb, Hassan Hosseini

**Affiliations:** 1 Faculty of Pharmacy, Al Rafidain University, Baghdad, Iraq; 2 School of Pharmacy, Monash University Malaysia, Bandar Sunway, Selangor, Malaysia; 3 Department of Public Health, Mohammed VI Center for Research and Innovation, Rabat, Morocco; 4 Mohammed VI International School of Public Health, Mohammed VI University of Sciences and Health, Casablanca, Morocco; 5 Higher Institute of Nursing Professions and Health Techniques, Ministry of Health and Social Protection, Rabat, Morocco; 6 St. Petersburg State Pediatric Medical University of the Ministry of Health of Russian Federation, Saint Petersburg, Russia; 7 Pharmacy Department, National Marrow Bone Transplant Center of Tunisia, Ariana, Tunisia; 8 Analytical Chemistry Department, Faculty of Pharmacy Monastir, Monastir, Tunisia; 9 Surgical Oncology Department, Oncology Center, Mansoura University, Mansoura, Egypt; 10 Occupational and Environmental Medicine, Community Medicine Department, Faculty of Medicine, Mansoura University, Mansoura, Egypt; 11 College of Pharmacy, Gulf Medical University, Ajman, United Arab Emirates; 12 Pharmacy Department, Sheikh Shakhbout Medical City, Abu Dhabi, United Arab Emirates; 13 Department of Clinical Pharmacy and Pharmacy Practice, Faculty of Pharmacy, University of Science and Technology, Sana’a, Yemen; 14 Head of Pediatric Oncology Department, National Oncology Center, Sana’a, Yemen; 15 Faculty of Medicine and Health Sciences, Hodeidah University, Al Hudaydah, Yemen; 16 Medical Oncology Department, Oncology Teaching Hospital Baghdad, Baghdad, Iraq; 17 Department of Public Health, Health Services Academy, Islamabad, Pakistan; 18 School of Pharmacy, Lebanese International University, Beirut, Lebanon; 19 Department of Biomedical Sciences, Lebanese International University, Beirut, Lebanon; 20 Center for Applied Mathematics and Bioinformatics (CAMB), Gulf University for Science and Technology, Kuwait City, Kuwait; 21 School of Medicine and Medical Sciences, Holy Spirit University of Kaslik, Jounieh, Lebanon; 22 Applied Science Research Center, Applied Science Private University, Amman, Jordan; 23 Paris-East Créteil University (UPEC)-University Paris-Est, Creteil, France; 24 RAMSAY SANTÉ, Hôpital Privé Paul d'Égine (HPPE), Champigny-sur-Marne, France

**Keywords:** attitude, chemotherapy, knowledge, oncologists, practice, resistance

## Abstract

**Introduction:**

This study assessed oncologists’ knowledge, attitudes, and practices regarding chemotherapy resistance across multiple countries.

**Methods:**

A cross-sectional study was conducted from September 2023 to February 2024 using a structured questionnaire adapted from previous literature and translated into French, Russian, and Chinese. Data were analyzed using SPSS. Independent t-tests, ANOVA, and multivariable logistic regression were used to examine associations between demographic characteristics and knowledge, attitude, and practice scores.

**Results:**

A total of 3,779 oncologists participated, with a mean age of 36.92 ± 9.24 years; 53.1% were female. Most participants were medical oncologists (50.0%), followed by clinical oncologists (23.4%) and radiation oncologists (18.8%). Nearly half had 0–9 years of professional practice, and the largest proportion were from Iraq (21.4%). In multivariable analysis, oncologists aged ≥56 years had lower odds of good knowledge (OR = 0.76, 95% CI: 0.58–0.98, p = 0.04). Female participants showed higher odds of good knowledge (OR = 1.22, 95% CI: 1.02–1.46, p = 0.03) and positive attitudes (OR = 1.20, 95% CI: 0.99–1.45, p = 0.04) than males. Post-doctorate holders also had greater odds of good knowledge (OR = 1.57, 95% CI: 1.25–1.97, p = 0.01) and positive attitudes (OR = 1.50, 95% CI: 1.20–1.87, p = 0.01) compared with those holding a medical bachelor’s degree. Longer professional experience, ≥10 years in the cancer field, and previous exposure to chemotherapy resistance cases were also associated with better knowledge and more positive attitudes.

**Conclusion:**

The findings highlight important gaps in oncologists’ knowledge and practices regarding chemotherapy resistance. Targeted educational programs and continuous professional development, particularly in underrepresented regions, may help strengthen clinical preparedness and improve chemotherapy resistance management.

## Introduction

Chemotherapy is a cornerstone in the treatment of cancer, offering curative and palliative benefits across a wide range of malignancies (1, 2). Despite its critical role, the efficacy of chemotherapy is increasingly undermined by the development of drug resistance, which is a leading cause of treatment failure and high mortality rate among cancer patients (3). The phenomenon of chemotherapy resistance has been observed in various cancers, including breast, lung, colorectal, and ovarian. It manifests through multiple mechanisms, including drug efflux, DNA damage repair, inhibition of cell death, epithelial-mesenchymal transition, and maintenance of cancer stem cells. These mechanisms often act in concert, leading to heterogeneous, adaptive resistance that complicates treatment and diminishes the long-term effectiveness of chemotherapy (4).

The clinical implications of chemotherapy resistance are profound. Patients with resistant tumors often experience disease progression despite receiving standard treatment regimens, necessitating more aggressive and often more toxic therapies (4–6). Additionally, the development of resistance can significantly reduce patients’ quality of life, requiring prolonged treatment courses, which are associated with detrimental side effects, increased hospitalizations, and higher healthcare costs (7). In the context of personalized medicine, there is growing evidence supporting the need to tailor cancer treatment not only to tumor characteristics but also to the likelihood of developing resistance to chemotherapy (7).

Despite the wealth of research on the molecular mechanisms of chemotherapy resistance, there is a critical gap in the literature regarding how oncologists perceive and manage this challenge in clinical practice (8). The knowledge, attitudes, and practices of oncologists play a crucial role in the effective management of chemotherapy resistance, as they are responsible for making complex treatment decisions that can significantly impact patient outcomes (8). Previous studies have highlighted variations in oncologists’ understanding of resistance mechanisms and discrepancies in confidence in managing resistant cases, attributed to geographical region, level of expertise, and institutional resources (9–11). For instance, oncologists in resource-limited settings may have limited access to advanced diagnostic tools that can detect early resistance leading to delayed change in treatment strategies and poor outcomes among cancer patients (12). Additionally, there is evidence that oncologists’ attitudes toward the use of alternative therapies, such as targeted agents or immunotherapy in the setting of resistance, vary widely, further complicating the management of resistant cases (8).

Given the global burden of cancer and the increasing incidence of chemotherapy resistance, it is imperative to identify the areas where knowledge is lacking across the various practice settings. The findings of this study could highlight the importance of targeted educational interventions and policy changes to improve the management of chemotherapy resistance. Furthermore, the study seeks to contribute to the broader discourse on the need for global strategies to combat chemotherapy resistance, particularly in the context of increasing access to personalized and precision medicine. Thus, this study aims to evaluate oncologists’ knowledge, attitudes, and practices across different regions and healthcare settings and to determine the influence of associated factors that significantly affect knowledge and attitudes.

## Methods

### Study design and study period

This cross-sectional study was conducted between September 2023 and February 2024 across multiple countries, including Morocco, Tunisia, Egypt, Yemen, the United Arab Emirates, Russia, Pakistan, Iraq, and China. These countries were strategically selected to represent a diverse range of healthcare systems, economic statuses, and cultural attitudes toward cancer treatment. The selection aimed to capture a broad spectrum of oncological practices and perspectives on chemotherapy resistance, providing insights that are both globally relevant and regionally specific. The study was designed to assess oncologists’ knowledge, attitudes, and practices regarding chemotherapy resistance. Oncologists were recruited from various healthcare settings, including public and private hospitals, specialized cancer treatment centers, and academic institutions, to ensure comprehensive coverage of the field of oncology. The inclusion criteria were practicing oncologists with at least 1 year of experience in oncology. Healthcare professionals who were not working in oncology were excluded from the study. The diverse geographic and clinical settings were chosen to provide a comprehensive understanding of how chemotherapy resistance is managed across different regions and healthcare environments. This study was reported in accordance with the Strengthening the Reporting of Observational Studies in Epidemiology (STROBE) guidelines.

### Study instrument and translation

The study utilized a structured questionnaire adapted from a previously validated tool used in similar research on chemotherapy resistance (8). The original questionnaire, developed in English, consisted of 72 items divided into four sections: 9 items on demographic characteristics, 32 on knowledge, 13 on attitudes, and 18 on practices related to chemotherapy resistance. Given the international scope of the study, the questionnaire was translated into French, Russian, and Chinese to ensure accessibility to participants from various linguistic backgrounds, as demonstrated in the **Supplementary Materials**.

The translation process involved several critical steps to ensure the accuracy and validity of the translated questionnaires. Initially, forward translation was performed, wherein the English questionnaire was translated into French, Russian, and Chinese by bilingual experts proficient in both the source and target languages and knowledgeable about medical terminology. This was followed by backward translation, in which multiple bilingual experts, unaware of the original questionnaire, translated the questionnaires back into English. This step was crucial in identifying any discrepancies between the original and translated versions.

Subsequently, the back-translated English versions were compared with the original questionnaire by a panel of oncology specialists fluent in both languages. This expert panel reviewed the translations for conceptual equivalence and cultural relevance, ensuring that the meaning of the questions was preserved across languages. To further refine the translations, the translated questionnaires were pre-tested with small groups of oncologists in each language group. Cognitive interviewing techniques were employed during this phase to evaluate the clarity and comprehension of the questions. Feedback from the pre-test participants was used to make necessary adjustments to the translations.

To confirm the validity and reliability of the translated questionnaires, several analytical tests were conducted. Content validity was assessed using the Content Validity Index (CVI), with scores ranging from 0.85 to 0.92 across the different language versions, indicating high content validity. Reliability was evaluated using Cronbach’s alpha, with scores of 0.89 for the French version, 0.87 for the Russian version, and 0.91 for the Chinese version, demonstrating strong internal consistency.

The questionnaire was adapted from previously validated KAP instruments reported in the literature (8). Internal consistency reliability testing demonstrated satisfactory Cronbach’s alpha coefficients ranging from 0.87 to 0.91.

### Sample size and sampling

The minimum required sample size was calculated using Cochran’s formula for large populations (1). The calculation assumed a 95% confidence level (Z = 1.96) and a margin of error (e) of 5% (0.05). Lacking prior specific data on the prevalence of chemotherapy resistance knowledge among oncologists in the target population, a conservative estimate of 50% (p=0.5) was used for the population proportion. This value maximizes the required sample size, thereby ensuring sufficient statistical power (13). Based on these parameters, the minimum sample size required per country was determined to be approximately 385 participants


(n=[Z^2*p*(1−p)]/[e^2]=[1.96^2*0.5*0.5]/[0.05^2]≈384.16)

Given that the study aimed for representation across 9 countries (Morocco, Tunisia, Egypt, Yemen, UAE, Russia, Pakistan, Iraq, and China), this calculation implied a minimum total sample size of approximately 3,465 participants (385×9) to achieve the desired precision.

A total of 5,500 oncologists were subsequently invited to participate in the study via email, professional networks, and social media platforms. Of these, 3,779 completed the survey, yielding an overall response rate of approximately 68.7%.

To reach a wide, diverse group of participants worldwide, a snowball sampling method was employed. This involved leveraging the professional networks of initial contacts identified by the research team and encouraging participants to forward the survey link to eligible colleagues. Social media platforms popular within the medical community were also utilized for dissemination. This combined approach aimed to maximize reach and enhance the sample’s representativeness within the global oncology community.

### Ethical approval and consent to participate

The study was conducted in accordance with the Declaration of Helsinki and approved by the Research Committee of Al Rafidain University College (REC-46-2023). All participants were provided with an information sheet detailing the study’s purpose, their role as participants, and their right to withdraw from the study at any point without penalty. Electronic informed consent was obtained from each participant before they could access the survey. The participants’ anonymity was ensured, and all data were stored securely and accessible only to the research team.

### Data collection

Data were collected using SurveyMonkey, an online survey platform, which facilitated efficient distribution and management of responses across the diverse geographic locations involved in the study. The survey link was distributed via email, social media, and professional networks, enhancing the study’s reach across different regions. To maximize the response rate, several strategies were employed, including multiple reminder emails and optimizing the survey design for ease of use. The survey was conducted anonymously, with no identifiable personal information collected. Participants were informed about the study’s purpose, the voluntary nature of participation, and the confidentiality of responses. Electronic informed consent was obtained before access was granted. The estimated completion time was 15–20 minutes. The questionnaire comprised four main sections (as detailed in the Study Instrument section): demographic characteristics, knowledge regarding chemotherapy resistance, attitudes toward its management, and current practices related to chemotherapy resistance. The chemotherapy resistance knowledge score was assessed by summing the number of correct responses to 32 statements. Correct responses were defined as selecting “strongly agree” or “agree” for true statements (e.g., “Chemotherapy resistance is a critical health issue worldwide”), or “strongly disagree” or “disagree” for false statements (e.g., “Every cancer patient treated with chemotherapy is at high risk of chemotherapy resistance”). Each correct response was awarded one point; incorrect or uncertain responses received zero points. The maximum score was 32, categorized into low (0–16), moderate (17–26), and high (27–32) based on established benchmarks (8). Attitude toward chemotherapy resistance management was evaluated by summing appropriate responses to 13 items. Appropriate responses were defined as “strongly agree” or “agree” for positive statements (e.g., “Chemotherapy resistance decreases the chance of survival”) and “strongly disagree” or “disagree” for negative statements (e.g., “Chemotherapy resistance is all because of the oncologist’s fault”). Each appropriate response earned one point, for a maximum score of 13. Scores were categorized as negative (0–6) or positive (7–13) according to validated methods. The fourth section included 18 questions that examined oncologists’ self-reported current practices regarding chemotherapy resistance. Unlike the knowledge and attitude sections, these practice-related questions were not combined into a single score because they were descriptive (e.g., asking about specific actions taken or the frequency of certain procedures). Data from this section were analyzed descriptively, focusing on the frequencies and percentages of responses to individual items to understand common patterns and variations in clinical practice (8).

### Data analysis

All statistical analyses were conducted using SPSS version 29.00. Continuous variables were presented as mean ± standard deviation and 95% confidence interval (CI). Categorical and ordinal variables were shown as frequencies (n) and percentages (%). Independent t-tests were used to compare mean knowledge and attitude scores between two groups (e.g., gender), while one-way ANOVA was used to compare scores across multiple groups (e.g., age categories and years of practice). Variables with a p < 0.2 in the bivariate analysis were included in the regression analysis. Multivariable logistic regression was conducted to examine associations between participant characteristics (independent variables) and knowledge and attitude scores (dependent variables). Results were presented as odds ratios (OR) and 95% CI. Statistical tests were two-tailed and reported statistically significant at p < 0.05. The practice domain was analyzed descriptively because the included items reflected heterogeneous clinical behaviors that were not considered suitable for generation of a unified composite score. Knowledge and attitude scores were categorized according to previously published KAP methodology to facilitate interpretation and comparison with prior studies.

## Results

### Sociodemographic characteristics of participants

A total of 3,779 oncologists were enrolled in the study, with a mean age of 36.92 ± 9.235, 53.1% were females. The majority were medical oncologists (50.0%), followed by chemotherapy oncologists (23.4%) and radiation oncologists (18.8%). Nearly half of the participants (50.0%) had 0-9 years of practice and were mainly from Iraq (21.4%). **Table 1** summarizes the sociodemographic characteristics of all the participants.

**Table 1:** Demographic characteristics of participants (n=3779).

Characteristic	N (%)
Age (Mean ± SD)	**36.92 ± 9.235**
26-35	2183 (57.8%)
36-45	945 (25.0%)
46-55	355 (9.4%)
≥ 56	296 (7.8%)
Gender	
Male	1772 (46.9%)
Female	2007 (53.1%)
Educational level	
Med Bachelor	1061 (28.1%)
Med Master	117 (3.1%)
Medical Doctor	1890 (50.0%)
Post Doctorate	711 (18.8%)
Clinical specialization	
Medical oncologist	1890 (50.0%)
Surgical oncologist	295 (7.8%)
Radiation oncologist	711 (18.8%)
Chemotherapy oncologist	883 (23.4%)
Total years in practice (mean ± SD = 11.39 ± 8.09)	
0-9	1889 (50.0%)
10-19	1417 (37.5%)
≥ 20	473 (12.5%)
Years in cancer field (mean ± SD = 6.72 ± 4.57)	
0-4	1544 (40.9%)
6-9	1062 (28.1%)
≥ 10	1173 (31.0%)
Title of position	
Professor	178 (4.7%)
Assistant Professor	1005 (26.6%)
Resident	2245 (59.4%)
Fellow	351 (9.3%)
Experienced chemotherapy resistance cases before	
Yes	2646 (70.0%)
No	1133 (30.0%)
Country	
Morocco	394 (10.4%)
Tunisia	607 (16.1%)
Egypt	404 (10.7%)
Yemen	729 (19.3%)
UAE (Local)	162 (4.3%)
UAE (Resident)	365 (9.7%)
Russia	426 (11.3%)
Pakistan	93 (2.5%)
Iraq	809 (21.4%)
China	190 (5.0%)

### Level of knowledge and attitude toward chemotherapy resistance

**Figure 1:**
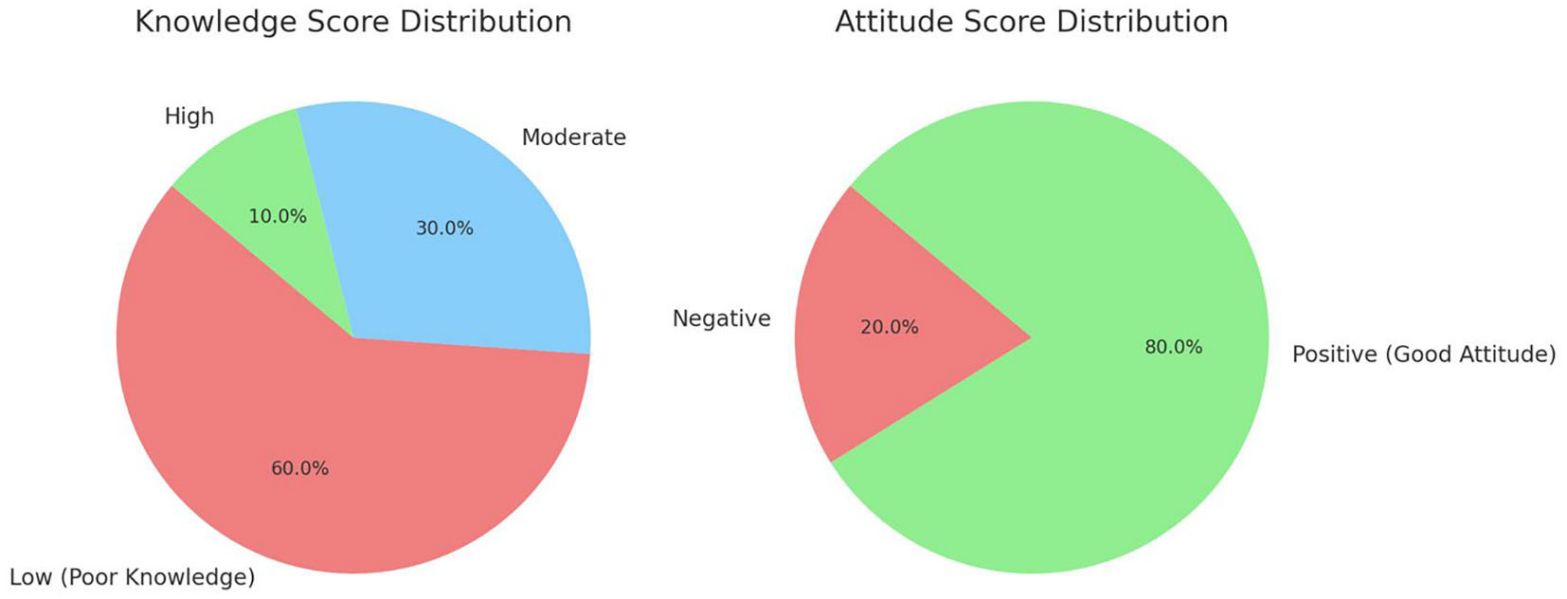
The level of knowledge and attitude of participants toward chemotherapy resistance.

**Figure 1** illustrates the distribution of knowledge and attitude levels among the participants regarding chemotherapy resistance. The majority of participants exhibited low knowledge levels, but displayed a positive attitude toward managing chemotherapy resistance (60% and 80%), respectively. This distribution suggests that while there is a significant knowledge gap among oncologists regarding chemotherapy resistance, the overall attitude toward addressing this issue remains predominantly positive.

### Oncologists’ practices toward chemotherapy resistance

**Figure 2:**
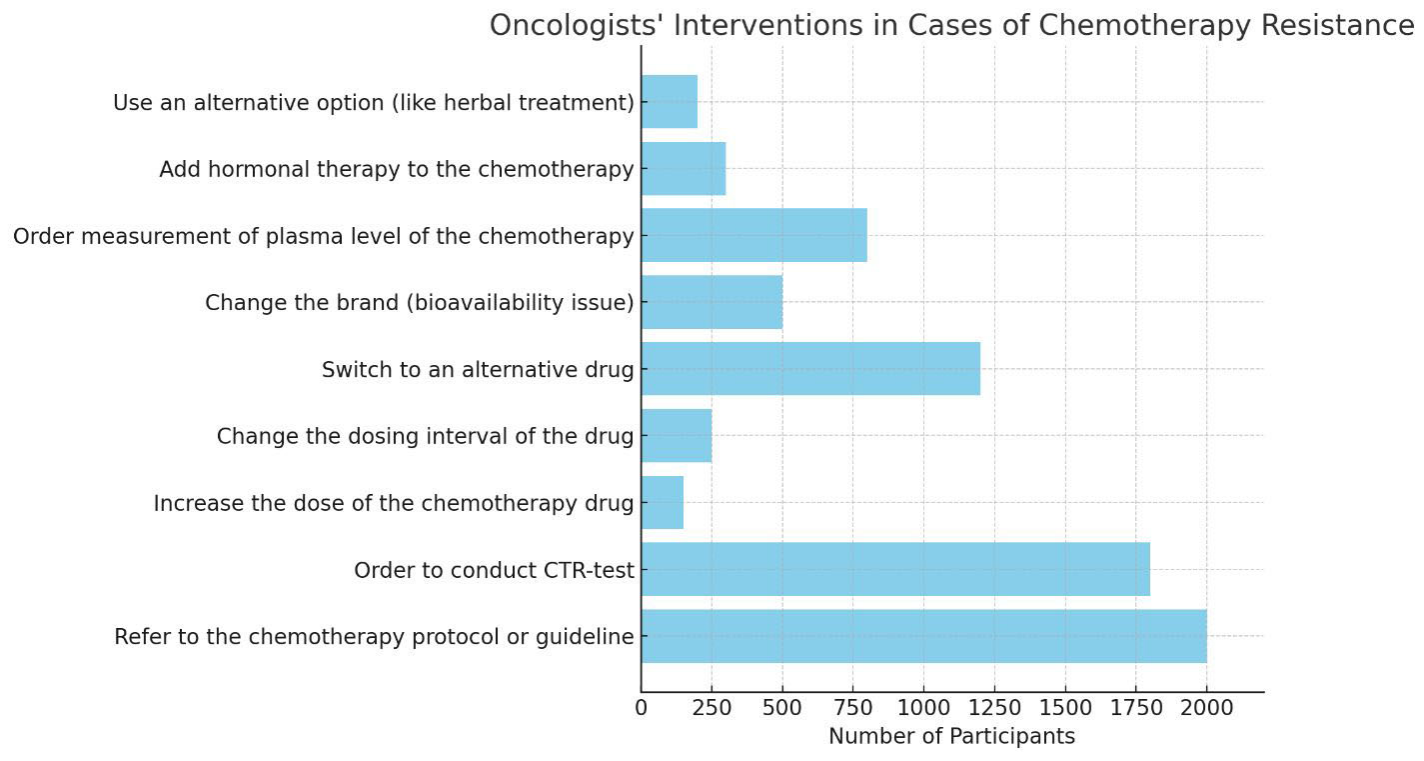
Oncologists’ actions toward chemotherapy resistance cases.

Common actions taken by oncologists when faced with chemotherapy resistance are shown in **Figure 2**. The majority of participants relied heavily on established protocols, with nearly 40% referring to chemotherapy guidelines and about 34% ordering Cancer Therapy Response-Test (CTR-Test^®^). Switching to alternative drugs was also a frequent strategy, adopted by 32% of the respondents. In contrast, conventional approaches, such as increasing the dose of chemotherapy or adding hormonal therapy, were reported by fewer participants, indicating these are reserved for more specific or challenging cases.

**Figure 3:**
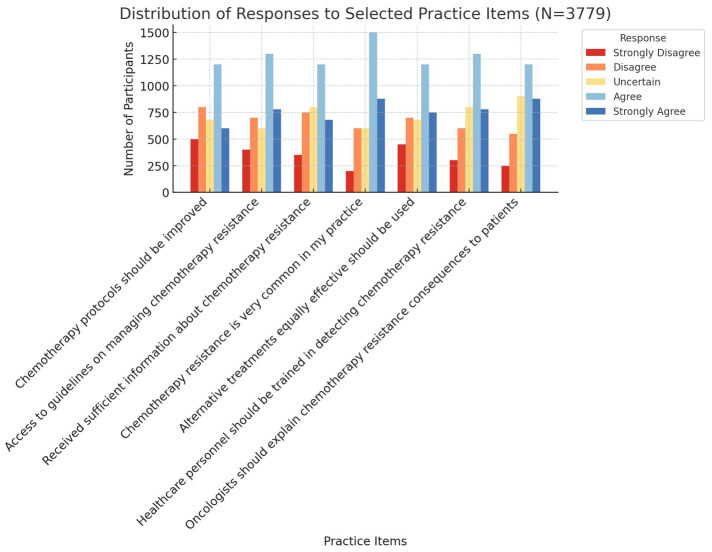
Oncologists’ practice toward chemotherapy resistance.

Moreover, a significant portion of oncologists (around 65%) supported the need for improved chemotherapy protocols, as indicated by access to necessary guidelines. Figure three also highlights areas of uncertainty or variability, particularly regarding the use of alternative treatments and the perception of chemotherapy resistance in clinical practice as shown in **Figure 3**. These findings suggest a commitment to improving practices but also underscore the challenges in achieving consistency across different settings.

### Differences in knowledge and attitude scores by participants’ demographic characteristics

**Table 2** reveals several notable differences in knowledge and attitude scores among oncologists based on their demographic characteristics. Among the key findings, oncologists with post-doctorate degrees scored notably higher in both knowledge and attitude, with mean scores of 23.4 and 10.2, respectively (p = 0.04 and p = 0.03). This suggests that higher levels of education are associated with greater knowledge and more positive attitudes toward chemotherapy resistance. Similarly, clinical specialization showed significant variation, with medical oncologists achieving higher mean knowledge (23.0) and attitude (10.0) scores than their counterparts in other specializations (p = 0.02 and p = 0.01, respectively). This reflects the specialized knowledge and expertise that medical oncologists possess regarding chemotherapy and its resistance. Furthermore, years of practice also played a significant role, as oncologists with more than 20 years of experience demonstrated higher knowledge and attitude scores, with 23.2 and 10.1, respectively (p = 0.03 and p = 0.02).

**Table 2:** Differences in knowledge and attitude scores by participants’ demographic characteristics (N = 3779).

Variable	Knowledge score mean (SD)	P-Value (Knowledge)	Attitude score mean (SD)	P-Value (Attitude)
Age				
26-35	22.1 (3.2)	0.10*	9.8 (2.1)	0.09*
36-45	23.0 (3.0)		10.0 (2.0)	
46-55	21.5 (3.4)		9.5 (2.2)	
≥ 56	20.9 (3.6)		9.2 (2.3)	
Gender				
Male	22.0 (3.3)	0.08#	9.7 (2.1)	0.07#
Female	23.2 (3.1)		10.1 (2.0)	
Educational level				
Med Bachelor	21.8 (3.5)	0.04*	9.4 (2.3)	0.03*
Med Master	22.5 (3.2)		9.9 (2.1)	
Medical Doctor	23.1 (3.0)		10.0 (2.0)	
Post Doctorate	23.4 (2.9)		10.2 (1.9)	
Clinical specialization				
Medical oncologist	23.0 (3.1)	0.02*	10.0 (2.0)	0.01*
Surgical oncologist	22.3 (3.3)		9.7 (2.1)	
Radiation oncologist	22.6 (3.2)		9.8 (2.1)	
Chemotherapy oncologist	21.7 (3.5)		9.5 (2.3)	
Total years in practice				
0-9	21.5 (3.4)	0.03*	9.4 (2.3)	0.02*
10-19	22.7 (3.1)		9.9 (2.1)	
≥ 20	23.2 (3.0)		10.1 (2.0)	
Years in cancer field				
0-4	21.6 (3.4)	0.04*	9.5 (2.3)	0.03*
6-9	22.8 (3.1)		9.9 (2.1)	
≥ 10	23.1 (3.0)		10.0 (2.0)	
Title of position				
Professor	23.5 (2.9)	0.01*	10.2 (1.9)	0.01*
Assistant Professor	23.0 (3.1)		10.0 (2.0)	
Resident	21.9 (3.4)		9.6 (2.2)	
Fellow	22.4 (3.3)		9.8 (2.1)	
Experienced chemotherapy resistance cases before				
Yes	22.7 (3.1)	0.04#	9.9 (2.1)	0.03#
No	21.8 (3.4)		9.5 (2.3)	
Country				
Morocco	22.5 (3.4)	0.02*	9.8 (2.3)	0.02*
Tunisia	22.3 (3.3)		9.7 (2.1)	
Egypt	23.1 (3.0)		10.0 (2.0)	
Yemen	21.5 (3.4)		9.6 (2.2)	
UAE (Local)	23.3 (3.2)		9.9 (2.1)	
UAE (Resident)	22.8 (3.5)		9.6 (2.3)	
Russia	23.9 (3.6)		9.9 (2.1)	
Pakistan	22.4 (3.3)		9.7 (2.1)	
Iraq	22.0 (3.1)		9.8 (2.0)	
China	22.9 (3.2)		9.9 (2.1)	

* P-value calculated using ANOVA., # P-value calculated using independent t-test.

Additionally, oncologists from Iraq had a mean knowledge score of 23.0 and an attitude score of 10.0, indicating potential regional differences in education and clinical practices related to chemotherapy resistance (p = 0.02 for both). These findings suggest that higher educational attainment, specialization in medical oncology, and extensive clinical experience are associated with better knowledge and more positive attitudes toward chemotherapy resistance. Moreover, regional differences point to the need for targeted interventions to address gaps in knowledge and attitudes across different countries.

### Multivariable analysis

The multivariable analysis showed that oncologists aged 56 years and above had significantly lower knowledge scores (OR = 0.76, 95% CI: 0.58-0.98, p = 0.04). As for gender, female oncologists had higher odds of better knowledge (OR = 1.22, 95% CI: 1.02-1.46, p = 0.03) and more positive attitudes (OR = 1.20, 95% CI: 0.99-1.45, p = 0.04) compared to males. Additionally, oncologists with a post-doctorate degree had higher odds of possessing greater knowledge (OR = 1.57, 95% CI: 1.25-1.97, p = 0.01) and more positive attitudes (OR = 1.50, 95% CI: 1.20-1.87, p = 0.01) compared to participants with a Medical Bachelor’s degree. Similarly, years of practice and experience in the cancer field were significant predictors, with those having over 20 years of practice and 10 or more years in the cancer field showing higher odds of better knowledge and more positive attitudes, with a p-value <0.05.

Finally, prior experience with chemotherapy resistance cases significantly influenced both knowledge and attitude scores, with oncologists lacking such experience having a lower knowledge score (OR = 0.73, 95% CI: 0.57-0.95, p = 0.02) and a positive attitude (OR = 0.76, 95% CI: 0.58-1.00, p = 0.03). These results highlight the key demographic factors associated with variations in oncologists’ knowledge and attitudes toward chemotherapy resistance. **Table 3** represents the logistic regression analysis.

**Table 3:** Logistic regression analysis of demographic characteristics on knowledge and attitude scores.

Variable	Coefficient (B)	Knowledge OR (95% CI for Exp(B))	P (Logistic Regression - Knowledge)	Coefficient (B)	Attitude OR (95% CI for Exp(B))	P (Logistic Regression - Attitude)
Age						
26-35 (Reference)	0.00	1.00	–	0.00	1.00	–
36-45	0.15	1.16 (0.95 - 1.42)	0.12	0.10	1.11 (0.90 - 1.36)	0.14
46-55	-0.20	0.82 (0.65 - 1.04)	0.09	-0.15	0.86 (0.68 - 1.09)	0.11
≥ 56	-0.28	0.76 (0.58 - 0.98)	0.04	-0.22	0.80 (0.62 - 1.03)	0.08
Gender						
Male (Reference)	0.00	1.00	-	0.00	1.00	-
Female	0.20	1.22 (1.02 - 1.46)	0.03	0.18	1.20 (0.99 - 1.45)	0.04
Educational level						
Med Bachelor (Reference)	0.00	1.00	–	0.00	1.00	–
Medical Master	0.12	1.13 (0.88 - 1.45)	0.11	0.10	1.11 (0.86 - 1.42)	0.13
Medical Doctor	0.35	1.42 (1.18 - 1.71)	0.01	0.30	1.35 (1.11 - 1.63)	0.02
Post Doctorate	0.45	1.57 (1.25 - 1.97)	0.01	0.40	1.50 (1.20 - 1.87)	0.01
Clinical specialization						
Medical oncologist (Reference)	0.00	1.00	-	0.00	1.00	-
Surgical oncologist	-0.10	0.90 (0.71 - 1.14)	0.12	-0.12	0.88 (0.69 - 1.12)	0.13
Radiation oncologist	0.08	1.08 (0.84 - 1.38)	0.15	0.10	1.11 (0.86 - 1.43)	0.14
Chemotherapy oncologist	-0.22	0.80 (0.62 - 1.03)	0.08	-0.20	0.82 (0.64 - 1.05)	0.09
Total years in practice						
0-9 (Reference)	0.00	1.00	–	0.00	1.00	–
10-19	0.22	1.25 (0.99 - 1.57)	0.06	0.20	1.22 (0.96 - 1.54)	0.07
≥ 20	0.28	1.32 (1.01 - 1.72)	0.03	0.26	1.30 (0.99 - 1.70)	0.04
Years in cancer field						
0-4 (Reference)	0.00	1.00	–	0.00	1.00	–
6-9	0.20	1.22 (0.98 - 1.52)	0.06	0.18	1.20 (0.96 - 1.49)	0.07
≥ 10	0.32	1.38 (1.07 - 1.77)	0.02	0.28	1.33 (1.03 - 1.72)	0.03
Title of position						
Professor (Reference)	0.00	1.00	–	0.00	1.00	–
Assistant Professor	-0.18	0.83 (0.65 - 1.08)	0.10	-0.15	0.86 (0.67 - 1.12)	0.11
Resident	-0.24	0.79 (0.61 - 1.03)	0.09	-0.22	0.81 (0.63 - 1.05)	0.10
Fellow	-0.14	0.87 (0.66 - 1.15)	0.12	-0.12	0.88 (0.67 - 1.17)	0.13
Experienced chemotherapy resistance cases before						
Yes (Reference)	0.00	1.00	-	0.00	1.00	-
No	-0.31	0.73 (0.57 - 0.95)	0.02	-0.27	0.76 (0.58 - 1.00)	0.03

## Discussion

This was the first study that employed a validated, multi-language questionnaire across multiple countries, which revealed critical gaps in knowledge, attitudes, and practices among oncologists regarding chemotherapy resistance. These findings align with the study’s objectives and provide valuable insights into the factors influencing oncologists’ preparedness to manage chemotherapy resistance effectively.

### Assessment of knowledge and attitude toward chemotherapy resistance

One of the key findings concerns variation in knowledge levels by age, with younger oncologists, particularly those in the 36–45 age group, demonstrating higher knowledge scores. This likely reflects the impact of more recent education and training, which may incorporate the latest advancements in chemotherapy resistance management. Previous research in oncology has shown that younger healthcare professionals are often more familiar with the latest treatment guidelines, including chemotherapy resistance, suggesting that continuous professional development is crucial for maintaining updated knowledge (13–15). This underscores the importance of ongoing education for all oncologists, particularly those who may have completed their formal training many years ago. Gender differences also emerged as a significant factor, with Female oncologists demonstrated higher knowledge and attitude scores in this sample; however, these associations may be influenced by unmeasured confounding factors such as professional experience, training exposure, and workplace characteristics. This trend is consistent with studies suggesting that female healthcare professionals are more engaged in continuing education and proactive in adopting new practices, including the management of chemotherapy resistance (8, 15, 16). These findings highlight the need for inclusive educational programs that encourage all oncologists to engage in continuous learning, regardless of gender. Also, higher educational exposure was associated with higher knowledge scores. Educational level played a crucial role, with oncologists holding postdoctoral degrees achieving the highest knowledge and attitude scores. This finding reinforces the critical role of advanced education in equipping oncologists with the necessary skills to manage chemotherapy resistance effectively. Continuous professional development, especially at advanced academic levels, has been shown to enhance clinical competencies, particularly in complex areas like chemotherapy resistance (17–19). This suggests that fostering opportunities for higher education could have a profound impact on improving oncology care globally.

### Evaluation of oncologists’ practice towards chemotherapy resistance

The study uncovered notable variability in how oncologists address chemotherapy resistance in their clinical routines. Oncologists with greater knowledge and positive attitudes were more likely to implement evidence-based practices to mitigate resistance, such as personalized treatment plans and regular monitoring of patient responses (20). This correlation aligns with existing literature, which emphasizes that informed and positive attitudes often translate into better clinical practices, especially in managing chemotherapy resistance8. However, a considerable proportion of oncologists reported limited incorporation of strategies to manage resistance, highlighting a gap between knowledge and practical application. Factors contributing to this gap may include institutional constraints, limited resources, or limited access to updated clinical guidelines specific to chemotherapy resistance (21, 22). Addressing these barriers is crucial to ensuring that improvements in knowledge and attitudes are effectively translated into clinical practice, ultimately enhancing patient outcomes.

Oncologists from regions such as Russia and the United Arab Emirates demonstrated higher knowledge scores and more consistent implementation of best practices for chemotherapy resistance. This variation may reflect the impact of localized training programs, access to resources, and differences in healthcare infrastructure. For instance, the higher scores observed among Russian oncologists may be attributed to robust continuing medical education programs that focus on chemotherapy resistance (23). Conversely, the lower scores and less consistent practices in regions like Yemen and Iraq have been dramatically affected by several wars, which may reflect challenges such as limited access to up-to-date information and healthcare resources, particularly in the area of chemotherapy resistance (24). These findings suggest that efforts to improve education and resource availability in underrepresented regions could help bridge these gaps and lead to more consistent global oncology practices.

Despite the generally low levels of knowledge, many oncologists displayed a positive attitude toward addressing chemotherapy resistance. This contrast between knowledge and attitude suggests that, while oncologists recognize the importance of this issue, there may be barriers preventing them from acquiring the necessary knowledge and applying it in practice, such as insufficient training opportunities or limited access to current information on chemotherapy resistance (5, 25, 26). This finding differs from other studies where knowledge, attitudes, and practices are more closely aligned, indicating a need for targeted educational interventions that address these specific barriers (8, 14).

### Strengths and limitations

This study has several strengths, including its international scope, the use of a multi-language, validated questionnaire, and the inclusion of a diverse sample of oncologists. These factors significantly enhance the generalizability of the findings and provide valuable insights into global oncology practices. Additionally, the detailed analysis of demographic factors offers a deeper understanding of the determinants of knowledge and attitudes toward chemotherapy resistance.

However, as with any research, some limitations should be considered. The cross-sectional design of this study limits the ability to establish causality between demographic factors and knowledge or attitude outcomes. The use of snowball sampling may have introduced selection bias and limited the representativeness of the study population. In addition, unequal distribution of participants across countries may have influenced the observed findings and limited the generalizability of the results. Country of practice was included as a covariate in regression analyses to partially account for inter-country variability. Although internal consistency reliability was strong, exploratory and confirmatory factor analyses were not performed, which may limit comprehensive assessment of construct validity across cultures. Categorization of continuous scores may have reduced statistical sensitivity and resulted in partial loss of information. While this design provides a snapshot of the current state of oncologists’ knowledge and attitudes, future longitudinal studies could build on these findings to explore causal relationships more effectively. The study also faced challenges related to the overrepresentation of certain regions, which could skew the results and limit the generalizability to other areas. Nevertheless, these findings highlight important regional disparities that can inform targeted interventions. The insights gained from this study could serve as a cornerstone for future research, guiding more extensive and region-specific studies that address these disparities in greater detail. Future studies may benefit from developing a standardized quantitative scoring system for practice-related items.

## Conclusion

This study reveals significant gaps and variability in oncologists’ knowledge, attitudes, and practices regarding chemotherapy resistance. These findings highlight the need for targeted educational interventions and resource allocation that consider specific demographic and regional factors to improve knowledge, attitudes, and practices leading to better management of chemotherapy resistance. By addressing these gaps, healthcare systems can better equip oncologists to implement effective strategies against chemotherapy resistance, ultimately improving patient outcomes.

## Data Availability

The raw data supporting the conclusions of this article will be made available by the authors, without undue reservation.

## References

[1] Acharya PC Kurosu M . Introduction to chemotherapy: general and clinical considerations. In: Medicinal chemistry of chemotherapeutic agents. Academic Press, Amsterdam (2023). p. 1–18.

[2] Melo-Alvim C Neves ME Santos JL Abrunhosa-Branquinho AN Barroso T Costa L . Radiotherapy, chemotherapy and immunotherapy current practice and future perspectives for recurrent/metastatic oral cavity squamous cell carcinoma. Diagnostics. (2023) 13:99. doi: 10.3390/diagnostics13010099 36611391 PMC9818309

[3] Wijnant GJ Dumetz F Dirkx L Bulté D Cuypers B Van Bocxlaer K . Tackling drug resistance and other causes of treatment failure in leishmaniasis. FrontTrop Dis. (2022) 3:837460. doi: 10.3389/fitd.2022.837460

[4] Dhanyamraju PK . Drug resistance mechanisms in cancers: execution of pro-survival strategies. J BioMed Res. (2024) 38:95–104. doi: 10.7555/JBR.37.20230082 38413011 PMC11001593

[5] Anand U Dey A Chandel AK Sanyal R Mishra A Pandey DK . Cancer chemotherapy and beyond: current status, drug candidates, associated risks and progress in targeted therapeutics. Genes Dis. (2023) 10:1367–401. doi: 10.1016/j.gendis.2022.07.014 37397557 PMC10310991

[6] Wang X Zhang H Chen X . Drug resistance and combating drug resistance in cancer. Cancer Drug Resist. (2019) 2:141–60. doi: 10.20517/cdr.2019.10 34322663 PMC8315569

[7] Krzyszczyk P Acevedo A Davidoff EJ Timmins LM Marrero-Berrios I Patel M . The growing role of precision and personalized medicine for cancer treatment. Technol (Singap World Sci). (2018) 6:79–100. doi: 10.1142/S2339547818300020 30713991 PMC6352312

[8] Hassan BA Mohammed AH Alsammarraie AZ Alabboodi MK Wayyes AM Ahmed AA . Knowledge, attitude, and practice of oncologists toward chemotherapy resistance: questionnaire development and pilot testing. Asian Pac J Cancer Prev. (2022) 23:4275–81. doi: 10.31557/APJCP.2022.23.12.4275 36580010 PMC9971488

[9] Lei ZN Tian Q Teng QX Wurpel JN Zeng L Pan Y . Understanding and targeting resistance mechanisms in cancer. MedComm. (2023) 4:e265. doi: 10.1002/mco2.265 37229486 PMC10203373

[10] Elmore LW Greer SF Daniels EC Saxe CC Melner MH Krawiec GM . Blueprint for cancer research: critical gaps and opportunities. CA Cancer J Clin. (2021) 71:107–39. doi: 10.3322/caac.21660 33326126

[11] Emran TB Shahriar A Mahmud AR Rahman T Abir MH Siddiquee MF . Multidrug resistance in cancer: understanding molecular mechanisms, immunoprevention and therapeutic approaches. Front Oncol. (2022) 12:891652. doi: 10.3389/fonc.2022.891652 35814435 PMC9262248

[12] Ramutumbu NJ Ramathuba DU Maputle MS . Barriers to accessing oncology services for effective cancer care in public health institutions in Limpopo Province, South Africa: a qualitative study. Nurs Rep. (2023) 13:956–68. doi: 10.3390/nursrep13030082 37489406 PMC10366909

[13] Adeoye MA . Review of sampling techniques for education. ASEAN J For Sci Educ. (2023) 2:87–94.

[14] Hays RB Ramani S Hassell A . Healthcare systems and the sciences of health professional education. Adv Health Sci Educ Theory Pract. (2020) 25:1149–68. doi: 10.1007/s10459-020-10010-1 33206272 PMC7672408

[15] Solera-Gómez S Benedito-Monleón A Llinares-Insa LI Sancho-Cantus D Navarro-Illana E . Educational needs in oncology nursing: a scoping review. Healthcare (Basel). (2022) 10:2494. doi: 10.3390/healthcare10122494 36554019 PMC9778242

[16] Jarva E Oikarinen A Andersson J Tuomikoski AM Kääriäinen M Meriläinen M . Healthcare professionals’ perceptions of digital health competence: a qualitative descriptive study. Nurs Open. (2022) 9:1379–93. doi: 10.1002/nop2.1160 35094493 PMC8859079

[17] Lim SA Khorrami A Wassersug RJ Agapoff JA . Gender differences among healthcare providers in the promotion of patient-, person- and family-centered care and its implications for quality healthcare. Healthcare (Basel). (2023) 11:565. doi: 10.3390/healthcare11040565 36833099 PMC9957388

[18] Chong WF Chua J Leong LZ Smith HE Yu KY . Proactive career management for female health professionals: a scoping review protocol. BMJ Open. (2023) 13:e062716. doi: 10.1136/bmjopen-2022-062716 36737080 PMC9899976

[19] Lively A Minard LV Scott S Deal H Lambourne T Giffin J . Exploring healthcare professionals’ perspectives in delivering optimal oncology medication education. PloS One. (2020) 15:e0228571. doi: 10.1371/journal.pone.0228571 32049970 PMC7015363

[20] Lau ST Siah CJ Loh WL Rusli KD Schmidt LT Lim FP . Enhancing professional competency in clinical procedures using head-mounted display virtual reality: a mixed-methods study. Med Educ Online. (2023) 28:2232134. doi: 10.1080/10872981.2023.2232134 37406175 PMC10324421

[21] Ramírez-Morera A Tristán M Salazar-Vargas J Rivera-Chavarría AL . Effects of evidence-based clinical practice guidelines for breast cancer on healthcare quality improvement: a systematic review. F1000Res. (2022) 11:1035. doi: 10.12688/f1000research.122344.1 PMC9780606 36619604

[22] Eccles SA Aboagye EO Ali S Anderson AS Armes J Berditchevski F . Critical research gaps and translational priorities for the successful prevention and treatment of breast cancer. Breast Cancer Res. (2013) 15:1–37. doi: 10.1186/bcr3493 24286369 PMC3907091

[23] Eslami M Memarsadeghi O Davarpanah A Arti A Nayernia K Behnam B . Overcoming chemotherapy resistance in metastatic cancer: a comprehensive review. Biomedicines. (2024) 12:183. doi: 10.3390/biomedicines12010183 38255288 PMC10812960

[24] Baron E Sittig M Kotov M Fomintsev I Gushchin V . Educational collaboration between Russian-born US physicians and Russian oncology trainees in evidence-based medicine. JCO Glob Oncol. (2021) 7:353–60. doi: 10.1200/GO.20.00546 33667114 PMC8081499

[25] Skelton M Al-Mash’hadani AK Abdul-Sater Z Saleem M Alsaad S Kahtan M . War and oncology: cancer care in five Iraqi provinces impacted by the ISIL conflict. Front Oncol. (2023) 13:1151242. doi: 10.3389/fonc.2023.1151242 37213303 PMC10196689

[26] Ward RA Fawell S Floc’h N Flemington V McKerrecher D Smith PD . Challenges and opportunities in cancer drug resistance. Chem Rev. (2021) 121:3297–351. doi: 10.1021/acs.chemrev.0c00383 32692162

