# Supplementary material for: Knowledge, attitudes, and practices toward chemotherapy resistance among oncologists: a multinational cross-sectional study
Source: Front Oncol. 2026 Jun 5;16:1815979. doi: 10.3389/fonc.2026.1815979 (PMC13278857; doi:10.3389/fonc.2026.1815979)
Supplement: Supplementary file 1 [file DataSheet1.docx]

**Chemotherapy Resistance among Oncologists: An International Cross-Sectional Study of Knowledge, Attitudes, and Practices**

Résistance à la chimiothérapie chez les oncologues : Étude transversale internationale sur les connaissances, attitudes et pratiques
Резистентность к химиотерапии среди онкологов: Международное поперечное исследование знаний, отношения и практик

肿瘤科医生的化疗耐药性：国际跨部门知识、态度和实践研究

**Sociodemographic Characteristics (Caractéristiques sociodémographiques) (Социодемографические характеристики) (社会人口特征)**

**1. Age (Âge) (Возраст) (年龄):**

______ years (années) (лет) (岁) *(write as numbers please)*

**2. Gender (Sexe) (Пол) (性别):**

- Male (Homme) (Мужской) (男)
- Female (Femme) (Женский) (女)

**3. Educational Level (Niveau d'éducation) (Уровень образования) (教育程度):**

- Medical Bachelor (Licence en sciences médicales) (Медицинский бакалавр) (医学学士)
- Medical Master (Master en sciences médicales) (Магистр медицины) (医学硕士)
- Medical Doctor (Docteur en médecine) (Врач) (医学博士)
- Post-doctorate (Post-doctorat) (Последипломное образование) (博士后)

**4. Clinical Specialization (Spécialisation clinique) (Клиническая специализация) (临床专业):**

- Medical oncologist (Médecin oncologue) (Медицинский онколог) (肿瘤内科医生)
- Surgical oncologist (Chirurgien oncologue) (Онколог-хирург) (肿瘤外科医生)
- Radiation oncologist (Oncologue radiothérapeute) (Онколог-радиотерапевт) (放射肿瘤学医生)
- Chemotherapy oncologist (Oncologue chimiothérapeute) (Онколог-химиотерапевт) (化疗肿瘤学医生)

**5. Years in practice as a doctor (Nombre d'années d'exercice en tant que médecin) (Количество лет врачебной практики) (作为医生的实践年限):**

______ years (années) (лет) (年)*)*

**6. Years of experience as an oncologist (Années d'expérience en tant qu'oncologue) (Количество лет онкологического стажа) (作为肿瘤科医生的工作经验):**

______ years (années) (лет) (年)

**7. Title or position (Titre ou poste) (Звание или должность) (职位或职称):**

- Professor (Professeur) (Профессор) (教授)
- Assistant Professor (Assistant Professeur) (Доцент) (助理教授)
- Resident (Résident) (Интерн) (住院医师)

**8. Have you experienced any chemotherapy resistance case before?**

Avez-vous déjà rencontré un cas de résistance à la chimiothérapie auparavant ?
Вы сталкивались ранее с каким-либо случаем резистентности к химиотерапии?您以前遇到过化疗耐药性病例吗？

- Yes (Oui) (Да) (是)
- No (Non) (Нет) (否)

**9. Country of Practice (Pays d'exercice en oncologie) (Страна, где вы работаете онкологом) (从事肿瘤科工作的国家):**

- Morocco (Maroc) (Марокко) (摩洛哥)
- Tunisia (Tunisie) (Тунис) (突尼斯)
- Egypt (Égypte) (Египет) (埃及)
- Yemen (Yémen) (Йемен) (也门)
- UAE (Local) (Émirats Arabes Unis) (ОАЭ) (阿联酋)
- Russia (Russie) (Россия) (俄罗斯)
- Pakistan (Pakistan) (Пакистан) (巴基斯坦)
- Iraq (Irak) (Ирак) (伊拉克)
- China (Chine) (Китай) (中国)

**Knowledge of oncologists toward chemotherapy resistance (Connaissance des oncologues sur la résistance à la chimiothérapie) (Знания онкологов о химиотерапевтической резистентности) (对化疗耐药性的了解)**

| **Items (Articles) (Предметы) (项目)** | **Strongly Disagree (Pas du tout d'accord) (Категорически не согласен) (完全不同意)** | **Disagree (Pas d'accord) (Не согласен) (不同意)** | **Uncertain (Incertain) (Не уверен) (不确定)** | **Agree (d’accord) (Согласен) (同意)** | **Strongly Agree (Tout à fait d'accord) (Полностью согласен) (完全同意)** |
| --- | --- | --- | --- | --- | --- |
| 1. I know what chemotherapy resistance is. (Je sais ce qu'est la résistance à la chimiothérapie) (Я знаю, что такое резистентность к химиотерапии). (我知道什么是化疗耐药性) |  |  |  |  |  |
| 2. I am aware that chemotherapy resistance can exist among cancer patients. (Je suis conscient(e) que la résistance à la chimiothérapie peut exister chez les patients atteints de cancer) (Я осведомлен(а), что среди пациентов с раком может существовать резистентность к химиотерапии). (我知道化疗耐药性可以存在于癌症患者中) |  |  |  |  |  |
| 3. I am aware that chemotherapy resistance can happen at any stage of cancer disease (Je suis conscient(e) que la résistance à la chimiothérapie peut survenir à n'importe quel stade de la maladie du cancer). (Я осведомлен(а), что резистентность к химиотерапии может возникать на любой стадии ракового заболевания). (我知道在癌症的任何阶段都可能发生化疗耐药性) |  |  |  |  |  |
| 4. I have sufficient knowledge about how to use chemotherapy appropriately for my current practice. (J'ai une connaissance suffisante sur la façon d'utiliser la chimiothérapie de manière appropriée dans ma pratique actuelle). (У меня достаточные знания о правильном применении химиотерапии для моей текущей практики) (我有足够的知识来适当使用化疗在我的当前实践中) |  |  |  |  |  |
| 5. I am aware of the activities and lifestyle habits that should be avoided to counter chemotherapy resistance. (Je suis conscient(e) des activités et des habitudes de vie à éviter pour contrer la résistance à la chimiothérapie). (Я осведомлен о деятельности и образе жизни для противодействия резистентности к химиотерапии). (我知道应该避免的活动和生活习惯以对抗化疗耐药性) |  |  |  |  |  |
| 6. I have sufficient knowledge about Chemotherapy-Resistance-Test (CRT-test). [J'ai une connaissance suffisante sur le test de résistance à la chimiothérapie (CRT-test)]. (У меня достаточные знания о тесте на резистентность к химиотерапии (CRT-тест). (我有足够的知识关于化疗耐药性测试 (CRT-test)) |  |  |  |  |  |
| 7. I used to ask for CTR-test prior to chemotherapy administration. (Avant l'administration de la chimiothérapie, j'avais l'habitude de demander un test de CTR). (Ранее я запрашивал о проведении теста на резистентность к химиотерапии (CTR-тест) перед началом химиотерапии). (在化疗管理之前我习惯于要求进行CTR测试) |  |  |  |  |  |
| 8. When CTR-test result showed slight resistance (SR), this means the treatment plan most likely would not lead to an effective chemotherapy. (Lorsqule résultat du test de CTR indiquait une légère résistance (SR), cela signifiait que le plan de traitement ne conduirait probablement pas à une chimiothérapie efficace). (Когда результаты CTR-теста показывали лёгкую степень резистентности (SR), это означало, что план лечения, скорее всего, не приведёт к эффективности химиотерапии). (当CTR测试结果显示轻微耐药性（SR）时，这意味着治疗计划很可能不会导致有效的化疗) |  |  |  |  |  |
| 9. When CTR-test result showed extreme resistance (ER), a new chemotherapy treatment should be planned. (Lorsque le résultat du test du CTR montre une résistance extrême (ER), un nouveau traitement de chimiothérapie devrait être envisagé). (При результатах CTR-теста, демонстрирующих экстремальную резистентность (ER), необходимо разработать новый план химиотерапии). (当CTR测试结果显示极端耐药性（ER）时，应该计划新的化疗方案) |  |  |  |  |  |
| 10. When CTR-test result showed medium resistance (MR), this means the probability of therapeutic failure is low. (Lorsque le résultat du test du CTR montre une résistance moyenne (MR), cela signifie que la probabilité d'échec thérapeutique est faible). (Когда результаты CTR-теста показывают среднюю резистентность (MR), это свидетельство низкой вероятности терапевтической неудачи). (当CTR测试结果显示中度耐药性（MR）时，这意味着治疗失败的概率较低) |  |  |  |  |  |
| 11. Chemotherapy resistance is a critical health issue worldwide. (La résistance à la chimiothérapie est un problème de santé critique à l'échelle mondiale). (Резистентность к химиотерапии - серьезная проблема для здоровья населения всего мира). (化疗耐药性是全球的一个重要健康问题) |  |  |  |  |  |
| 12. Chemotherapy resistance can be transmitted from one cancer patient to another. (La résistance à la chimiothérapie peut être transmise d'un patient atteint de cancer à un autre). (Резистентность к химиотерапии может передаваться от одного онкологического пациента другому). (化疗耐药性可以从一个癌症患者传给另一个患者) |  |  |  |  |  |
| 13. Every cancer patient treated with chemotherapy is at high risk of chemotherapy resistance. (Chaque patient atteint de cancer traité par chimiothérapie présente un risque élevé de développer une résistance à la chimiothérapie). (Каждый онкологический пациент, которому проводится химиотерапия, находится в высоком риске развития резистентности к химиотерапии). (每一个接受化疗的癌症患者都处于高风险的化疗耐药性中) |  |  |  |  |  |
| 14. Misuse of chemotherapy can lead to chemotherapy resistance. (Une mauvaise utilisation de la chimiothérapie peut conduire à une résistance à la chimiothérapie). (Неправильное использование химиотерапии может привести к развитию резистентности к ней). (误用化疗可以导致化疗耐药性) |  |  |  |  |  |
| 15. Chemotherapy resistance occurs when cancer cells become resistant to chemotherapy, and they no longer work as well. (La résistance à la chimiothérapie se produit lorsque les cellules cancéreuses deviennent résistantes à la chimiothérapie, et qu'elles ne fonctionnent plus aussi bien). (Резистентность к химиотерапии возникает, когда раковые клетки становятся устойчивыми к химиотерапии и перестают эффективно на нее реагировать). (化疗耐药性发生在癌细胞对化疗产生耐药性时，并且它们的效果不再像以前那样好) |  |  |  |  |  |
| 16. Chemotherapy resistance occurs because of a gene-mutation happened in the cancer tissue. (La résistance à la chimiothérapie survient en raison d'une mutation génétique survenue dans les tissus cancéreux). (Резистентность к химиотерапии возникает из-за мутации генов в опухолевой ткани). (化疗耐药性发生是因为癌组织中的基因突变) |  |  |  |  |  |
| 17. Chemotherapy resistance occurs because of cancer cells may pump the drug out of the cell as fast as it is going in using a molecule called p-glycoprotein. (La résistance à la chimiothérapie survient lorsque les cellules cancéreuses peuvent éliminer le médicament aussi rapidement qu'il entre dans la cellule en utilisant une molécule appelée la glycoprotéine P). (Резистентность к химиотерапии возникает из-за того, что раковые клетки могут выкачивать препарат из клетки так же быстро, как он попадает в нее, с использованием молекулы п-гликопротеин). (化疗耐药性发生是因为癌细胞可能使用一种称为p-糖蛋白的分子将药物从细胞中排出，其速度与药物进入细胞的速度一样快) |  |  |  |  |  |
| Items (Articles) (Предметы) (项目) | Strongly Disagree (Pas du tout d'accord) (Категорически не согласен) (完全不同意) | Disagree (Pas d'accord) (Не согласен) (不同意) | Uncertain (Incertain) (Не уверен) (不确定) | Agree (d’accord) (Согласен) (同意) | Strongly Agree (Tout à fait d'accord) (Полностью согласен) (完全同意) |
| 18. Chemotherapy resistance occurs because of cancer cells may stop taking in the drugs because the protein that transports the drug across the cell wall stops working. (La résistance à la chimiothérapie survient lorsque les cellules cancéreuses arrêtent d'absorber les médicaments car la protéine qui transporte le médicament à travers la paroi cellulaire cesse de fonctionner). (Резистентность к химиотерапии возникает из-за того, что раковые клетки могут перестать впускать препараты, так как белок, отвечающий за транспорт препарата через клеточную стенку, перестает функционировать). (化疗耐药性发生是因为癌细胞可能停止摄取药物，因为运输药物穿过细胞壁的蛋白质停止工作) |  |  |  |  |  |
| 19. Chemotherapy resistance occurs because of the cancer cells may learn how to repair the DNA breaks caused by some anti-cancer drugs. (La résistance à la chimiothérapie survient lorsque les cellules cancéreuses apprennent à réparer les cassures de l'ADN causées par certains médicaments anticancéreux). (Резистентность к химиотерапии возникает из-за того, что раковые клетки могут научиться восстанавливать повреждения ДНК, вызванные некоторыми противораковыми препаратами). (化疗耐药性发生是因为癌细胞可能学会修复某些抗癌药物引起的DNA断裂) |  |  |  |  |  |
| 20. Chemotherapy resistance occurs because of cancer cells may develop a mechanism that inactivates the drug. (La résistance à la chimiothérapie survient lorsque les cellules cancéreuses développent un mécanisme qui inactive le médicament). (Резистентность к химиотерапии возникает из-за того, что раковые клетки могут развить механизм инактивации химиопрепарата). (化疗耐药性发生是因为癌细胞可能会开发一种使药物失活的机制) |  |  |  |  |  |
| 21. Chemotherapy resistance occurs because of some of the cancer cells that are not killed by the chemotherapy mutate (change) and become resistant to the drug. (La résistance à la chimiothérapie se produit en raison de certaines cellules cancéreuses qui ne sont pas tuées par la chimiothérapie, elles mutent (changent) et deviennent résistantes au médicament). (Резистентность к химиотерапии возникает из-за того, что некоторые раковые клетки, которые не уничтожены химиотерапией, мутируют и становятся устойчивыми к химиопрепарату). (化疗耐药性发生是因为某些未被化疗杀死的癌细胞突变（改变）并对药物产生抗药性) |  |  |  |  |  |
| 22. Delaying chemotherapy plays a major role in incidence of chemotherapy resistance. (Le retard de la chimiothérapie joue un rôle majeur dans l'incidence de la résistance à la chimiothérapie). (Задержка начала химиотерапии играет важную роль в процессе развития резистентности к химиотерапии). (推迟化疗在化疗耐药性发生中起主要作用) |  |  |  |  |  |
| 23. Reducing chemotherapy dose plays a major role in incidence of chemotherapy resistance. (La réduction de la dose de chimiothérapie joue un rôle majeur dans l'incidence de la résistance à la chimiothérapie). (Снижение дозы химиотерапии играет важную роль в возникновении резистентности к химиотерапии). (减少化疗剂量在化疗耐药性发生中起主要作用) |  |  |  |  |  |
| 24. Using of mono-chemotherapy rather than combination-chemotherapy plays a major role in incidence of chemotherapy resistance. (L'utilisation d'une chimiothérapie mono plutôt que d'une chimiothérapie combinée joue un rôle majeur dans l'incidence de la résistance à la chimiothérapie). (Использование монотерапии вместо комбинированной химиотерапии играет важную роль в возникновении резистентности к химиотерапии). (使用单一化疗而不是组合化疗在化疗耐药性发生中起主要作用) |  |  |  |  |  |
| 25. Chemotherapy resistance is associated with the use of specific types of chemotherapy. (La résistance à la chimiothérapie est associée à l'utilisation de certains types spécifiques de chimiothérapie). (Резистентность к химиотерапии связана с использованием определенных типов химиотерапии). (化疗耐药性与使用特定类型的化疗有关) |  |  |  |  |  |
| 26. The use or administration of multiple types of chemotherapy treatments can cause chemotherapy resistance. (L'utilisation ou l'administration de plusieurs types de traitements de chimiothérapie peut entraîner une résistance à la chimiothérapie). (Использование или применение нескольких типов химиотерапии может вызвать резистентность к химиотерапии). (使用或施用多种化疗方案可能导致化疗耐药性) |  |  |  |  |  |
| 27. Long duration (i.e., number of cycles) of chemotherapy can cause chemotherapy resistance. (Une durée prolongée (c'est-à-dire le nombre de cycles) de chimiothérapie peut causer une résistance à la chimiothérapie). (Длительная продолжительность курсов химиотерапии может вызвать резистентность к химиотерапии). (长期化疗（即周期数）可能导致化疗耐药性) |  |  |  |  |  |
| 28. High dose of chemotherapy can cause chemotherapy resistance. (Une dose élevée de chimiothérapie peut causer une résistance à la chimiothérapie). (Высокая доза химиотерапии может вызвать резистентность к химиотерапии). (高剂量化疗可能导致化疗耐药性) |  |  |  |  |  |
| 29. Route of chemotherapy administration can play role in incidence of chemotherapy resistance. (La voie d'administration de la chimiothérapie peut jouer un rôle dans l'incidence de la résistance à la chimiothérapie). (Метод введения химиотерапии может играть роль в возникновении резистентности к химиотерапии). (化疗给药途径在化疗耐药性发生中起作用) |  |  |  |  |  |
| 30. Anemia plays a major role in incidence of chemotherapy resistance. (L'anémie joue un rôle majeur dans l'incidence de la résistance à la chimiothérapie). (Анемия играет важную роль в возникновении резистентности к химиотерапии). (贫血在化疗耐药性发生中起主要作用) |  |  |  |  |  |
| 31. Hormonal factors contribute to chemotherapy resistance in breast cancer. (Les facteurs hormonaux contribuent à la résistance à la chimiothérapie dans le cas du cancer du sein). (Гормональные факторы способствуют резистентности к химиотерапии при раке молочной железы). (激素因素有助于乳腺癌的化疗耐药性) |  |  |  |  |  |
| 32. Age is a determinant of chemotherapy resistance. (L'âge est un déterminant de la résistance à la chimiothérapie). (Возраст является фактором, влияющим на резистентность к химиотерапии). (年龄是化疗耐药性的决定因素) |  |  |  |  |  |

**Attitude of oncologists toward chemotherapy resistance** (Attitude des oncologues face à la résistance à la chimiothérapie) (Отношение онкологов к резистентности к химиотерапии) (肿瘤科医生对化疗耐药性的态度)

| **Items (Articles) (Предметы) (项目)** | **Strongly Disagree (Pas du tout d'accord) (Категорически не согласен) (完全不同意)** | **Disagree (Pas d'accord) (Не согласен) (不同意)** | **Uncertain (Incertain) (Не уверен) (不确定)** | **Agree (d’accord) (Согласен) (同意)** | **Strongly Agree (Tout à fait d'accord) (Полностью согласен) (完全同意)** |
| --- | --- | --- | --- | --- | --- |
| 1. Chemotherapy resistance is the patient's fault. (La résistance à la chimiothérapie est la faute du patient). (Резистентность к химиотерапии - это вина пациента). (化疗耐药性是患者的错) |  |  |  |  |  |
| 2. Chemotherapy resistance is all because of the oncologist's fault. (La résistance à la chimiothérapie est entièrement due à la faute de l'oncologue). (Резистентность к химиотерапии - это полностью вина онколога). (化疗耐药性完全是肿瘤科医生的错) |  |  |  |  |  |
| 3. If proper steps are taken, chemotherapy resistance can be combated. (Si les mesures appropriées sont prises, la résistance à la chimiothérapie peut être combattue). (Если предпринять правильные шаги, резистентность к химиотерапии можно преодолеть). (如果采取适当措施，可以对抗化疗耐药性) |  |  |  |  |  |
| 4. I am confident that we can still beat cancer disease successfully after incidence of chemotherapy resistance. (Je suis convaincu que nous pouvons toujours vaincre le cancer après l'incidence de la résistance à la chimiothérapie). (Я уверен, что мы всё ещё можем успешно победить рак после появления резистентности к химиотерапии). (我相信在出现化疗耐药性后，我们仍然可以成功战胜癌症) |  |  |  |  |  |
| 5. If a patient receives proper information about the risk of chemotherapy resistance, it can be avoided. (Si un patient reçoit des informations appropriées sur le risque de résistance à la chimiothérapie, il peut l'éviter). (Если пациент получает правильную информацию о риске развития резистентности к химиотерапии, этого можно избежать). (如果患者收到有关化疗耐药风险的正确信息，就可以避免) |  |  |  |  |  |
| 6. Oncologists should fully assess factors that cause chemotherapy resistance in patients before commencing chemotherapy treatment. (Les oncologues doivent évaluer pleinement les facteurs qui causent la résistance à la chimiothérapie chez les patients avant de commencer le traitement de chimiothérapie). (Онкологи должны полностью оценить факторы, вызывающие резистентность к химиотерапии у пациентов, перед началом лечения). (肿瘤科医生应在开始化疗前充分评估导致患者化疗耐药性的因素) |  |  |  |  |  |
| 7. I am satisfied with the current treatment guideline to treat chemotherapy resistance. (Je suis satisfait des directives de traitement actuelles pour traiter la résistance à la chimiothérapie). (Я удовлетворен текущими рекомендациями по лечению резистентности к химиотерапии). (我对当前治疗化疗耐药性的指南感到满意) |  |  |  |  |  |
| 8. Rapid and effective diagnostic techniques are required for diagnosis of chemotherapy resistance. (Des techniques diagnostiques rapides et efficaces sont nécessaires pour diagnostiquer la résistance à la chimiothérapie). (Для диагностики резистентности к химиотерапии требуются быстрые и эффективные диагностические методы). (需要快速有效的诊断技术来诊断化疗耐药性) |  |  |  |  |  |
| 9. Personalizing chemotherapy treatment will help to prevent and/or overcome chemotherapy resistance problem. (La personnalisation du traitement de la chimiothérapie aidera à prévenir et/ou à surmonter le problème de la résistance à la chimiothérapie). (Персонализация химиотерапии поможет предотвратить и/или преодолеть проблему резистентности к химиотерапии). (个性化化疗治疗将有助于预防和/或克服化疗耐药性问题) |  |  |  |  |  |
| 10. National and healthcare policies are impractical against chemotherapy resistance. (Les politiques nationales et de santé sont impraticables contre la résistance à la chimiothérapie). (Национальная и медицинская политика непрактична в отношении резистентности к химиотерапии). (国家和卫生政策对化疗耐药性不切实际) |  |  |  |  |  |
| 11. Some chemotherapy resistance stems from genetic factors and it cannot be rectified. (Certaines résistances à la chimiothérapie proviennent de facteurs génétiques et ne peuvent être corrigées). (Некоторые резистентности к химиотерапии обусловлены генетическими факторами и их невозможно исправить). (一些化疗耐药性源于遗传因素，无法纠正) |  |  |  |  |  |
| 12. The patients’ coping mechanisms have a bearing on their ability to overcome drug resistance. (Les mécanismes d'adaptation des patients influent sur leur capacité à surmonter la résistance aux médicaments). (Механизмы преодоления пациентами влияют на их способность преодолевать лекарственную резистентность). (患者的应对机制对他们克服药物耐药性的能力有影响) |  |  |  |  |  |
| 13. Chemotherapy resistance decreases the chance of survival. (La résistance à la chimiothérapie diminue les chances de survie). (Резистентность к химиотерапии снижает шансы на выживание). (化疗耐药性降低生存机会) |  |  |  |  |  |

**Practice of oncologists toward chemotherapy resistance** (Pratique des oncologues face à la résistance à la chimiothérapie) (Практика онкологов в отношении резистентности к химиотерапии) (肿瘤科医生对化疗耐药性的实践)

| **Items (Articles) (Предметы) (项目)** | **Strongly Disagree (Pas du tout d'accord) (Категорически не согласен) (完全不同意)** | **Disagree (Pas d'accord) (Не согласен) (不同意)** | **Uncertain (Incertain) (Не уверен) (不确定)** | **Agree (d’accord) (Согласен) (同意)** | **Strongly Agree (Tout à fait d'accord) (Полностью согласен) (完全同意)** |
| --- | --- | --- | --- | --- | --- |
| 1. Chemotherapy protocols should be improved. (Les protocoles de chimiothérapie doivent être améliorés). (Протоколы химиотерапии должны быть улучшены). (化疗方案应予改进) |  |  |  |  |  |
| 2. I have easy access to guidelines I need on managing chemotherapy resistance. (J'ai un accès facile aux lignes directrices dont j'ai besoin pour gérer la résistance à la chimiothérapie). (У меня есть лёгкий доступ к руководствам, которые мне нужны для управления резистентностью к химиотерапии). (我可以轻松获取管理化疗耐药性的指南) |  |  |  |  |  |
| 3. I have easy access to the materials I need to give advice on prudent chemotherapy use and chemotherapy resistance. (J'ai un accès facile aux matériaux dont j'ai besoin pour donner des conseils sur l'utilisation prudente de la chimiothérapie et la résistance à la chimiothérapie). (У меня есть лёгкий доступ к материалам, которые мне нужны для предоставления рекомендаций по рациональному использованию химиотерапии и резистентности к ней). (我可以轻松获取提供化疗合理使用和化疗耐药性建议所需的材料) |  |  |  |  |  |
| 4. In the last 12 months, I received sufficient information about chemotherapy resistance. (Au cours des 12 derniers mois, j'ai reçu suffisamment d'informations sur la résistance à la chimiothérapie). (За последние 12 месяцев я получил достаточно информации о резистентности к химиотерапии). (在过去的12个月里，我获得了足够的关于化疗耐药性的信息) |  |  |  |  |  |
| 5. On the basis of information I receive, I change my practice on prescribing and/or administering of chemotherapy. (Sur la base des informations que je reçois, je change ma pratique en matière de prescription et/ou d'administration de la chimiothérapie). (На основании полученной информации я изменяю свою практику назначения и/или проведения химиотерапии). (根据我获得的信息，我改变了我的化疗处方和/或管理实践) |  |  |  |  |  |
| 6. My country has a national action plan on chemotherapy resistance. (Mon pays a un plan d'action national sur la résistance à la chimiothérapie). (В моей стране есть национальный план действий по борьбе с резистентностью к химиотерапии). (我国有一个针对化疗耐药性的国家行动计划) |  |  |  |  |  |
| 7. Chemotherapy resistance is very common in my practical settings. (La résistance à la chimiothérapie est très courante dans mes pratiques). (Резистентность к химиотерапии очень распространена в моей практике). (在我的实践中，化疗耐药性非常普遍) |  |  |  |  |  |
| 8. I usually consult my colleagues about any case of chemotherapy resistance. (Je consulte généralement mes collègues pour tout cas de résistance à la chimiothérapie). (Я обычно консультируюсь с коллегами по любому случаю резистентности к химиотерапии). (我通常会就任何化疗耐药性病例咨询我的同事) |  |  |  |  |  |
| 9. When there is chemotherapy resistance, alternative treatments, which are equally effective to chemotherapy, should be used. (Lorsqu'il y a une résistance à la chimiothérapie, des traitements alternatifs, aussi efficaces que la chimiothérapie, devraient être utilisés). (При возникновении резистентности к химиотерапии следует использовать альтернативные методы лечения, которые так же эффективны, как и химиотерапия). (当出现化疗耐药性时，应该使用与化疗同样有效的替代治疗方法) |  |  |  |  |  |
| 10. The sharing of information with patients on how to self-monitor and self-manage during chemotherapy resistance could be improved. (Le partage d'informations avec les patients sur la façon de s'autosurveillance et de s'autogérer pendant la résistance à la chimiothérapie pourrait être amélioré). (Информирование пациентов о том, как проводить самоконтроль и самоуправление во время резистентности к химиотерапии, может быть улучшено). (在化疗耐药性期间与患者分享自我监测和自我管理信息的方式可以改进) |  |  |  |  |  |
| 11. I treat a patient with chemotherapy resistance similarly to those who do not have chemotherapy resistance. (Je traite un patient présentant une résistance à la chimiothérapie de la même manière que ceux qui n'ont pas de résistance à la chimiothérapie). (Я лечу пациента с резистентностью к химиотерапии так же, как и тех, кто не имеет резистентности). (我像对待那些没有化疗耐药性的患者一样对待那些有化疗耐药性的患者) |  |  |  |  |  |
| 12. Incidence of chemotherapy resistance is more common in specific types of cancers than others. (L'incidence de la résistance à la chimiothérapie est plus fréquente dans certains types spécifiques de cancers que dans d'autres). (Частота возникновения резистентности к химиотерапии чаще встречается при некоторых типах рака, чем при других). (化疗耐药性在某些特定类型的癌症中比其他癌症更为常见) |  |  |  |  |  |
| 13. Cross-resistance in chemotherapy can be detected and rectified in patients. (La résistance croisée à la chimiothérapie peut être détectée et corrigée chez les patients). (Скрещенная резистентность при химиотерапии может быть выявлена и устранена у пациентов). (可以在患者中检测并纠正化疗中的交叉耐药性) |  |  |  |  |  |
| 14. Chemotherapy should be stopped immediately when it shows no signs of efficacy. (La chimiothérapie doit être arrêtée immédiatement lorsqu'elle ne montre aucun signe d'efficacité). (Химиотерапия должна быть немедленно прекращена, если она не проявляет признаков эффективности). (当化疗没有显示出有效的迹象时，应立即停止化疗) |  |  |  |  |  |
| 15. Physicians, nurses, and healthcare personnel should be trained in detecting chemotherapy resistance. (Les médecins, les infirmières et le personnel de santé doivent être formés à la détection de la résistance à la chimiothérapie). (Врачи, медсестры и медицинский персонал должны быть обучены выявлению резистентности к химиотерапии). (医生、护士和医护人员应接受化疗耐药性检测的培训) |  |  |  |  |  |
| 16. Oncologists should clearly explain to their patients about the consequences of chemotherapy resistance. (Les oncologues doivent expliquer clairement à leurs patients les conséquences de la résistance à la chimiothérapie). (Онкологи должны чётко объяснять своим пациентам последствия резистентности к химиотерапии). (肿瘤科医生应明确向患者解释化疗耐药性的后果) |  |  |  |  |  |
| 17. Therapeutic privilege among oncologists should be revised to allow more transparency. (Le privilège thérapeutique parmi les oncologues devrait être révisé pour permettre une plus grande transparence). (Терапевтический привилегий среди онкологов следует пересмотреть для обеспечения большей прозрачности). (应修改肿瘤科医生的治疗特权，以便更加透明) |  |  |  |  |  |

**18. While facing a case of chemotherapy resistance, I will: (can choose more than one answer):**

1. Face à un cas de résistance à la chimiothérapie, je vais : (peut choisir plus d'une réponse) : При столкновении с случаем резистентности к химиотерапии я буду: (можно выбрать более одного варианта ответа): 面对化疗耐药性案例时，我会：（可选择多个答案）：

a. Se référer au protocole ou à la ligne directrice de la chimiothérapie
Обратиться к протоколу или руководству по химиотерапии
参考化疗方案或指南

b. Ordonner la réalisation d'un test CTR
Назначить проведение CTR-теста
下令进行CTR测试

c. Augmenter la dose du médicament de chimiothérapie en cas de réponse inadéquate
Увеличить дозу химиотерапевтического препарата при недостаточной реакции
在没有足够反应的情况下增加化疗药物的剂量

d. Modifier l'intervalle de dosage du médicament (fréquence d'administration)
Изменить интервал дозирования препарата (частоту введения)
改变药物的剂量间隔（给药频率）

e. Changer complètement de médicament et passer à un médicament alternatif
Полностью сменить препарат и перевести пациента на альтернативный препарат
完全更换药物，将患者换到另一种药物

f. Garder le même médicament, mais changer la marque (problème de biodisponibilité)
Оставить тот же препарат, но изменить бренд (проблема с биодоступностью)
保留相同的药物，但更换品牌（生物利用度问题）

g. Ordonner la mesure du taux plasmatique de la chimiothérapie
Назначить измерение уровня химиотерапевтического препарата в плазме
下令测量化疗药物的血浆水平

h. Ajouter une thérapie hormonale au traitement de chimiothérapie
Добавить гормональную терапию к лечению химиотерапией
在化疗治疗中加入激素治疗

i. Arrêter le traitement de chimiothérapie et utiliser une option alternative (comme un traitement à base de plantes)
Прекратить лечение химиотерапией и использовать альтернативный вариант (например, травяное лечение)
停止化疗治疗，使用替代方案（如草药治疗）
