# Supplementary material for: Knowledge, attitudes, and practices toward chemotherapy resistance among oncologists: a multinational cross-sectional study
Source: Front Oncol. 2026 Jun 5;16:1815979. doi: 10.3389/fonc.2026.1815979 (PMC13278857; doi:10.3389/fonc.2026.1815979)
Supplement: Supplementary file 2 [file Table1.docx]

Appendix

Supplementary

**S1: Practice of Oncologists Toward Chemotherapy Resistance (n=3779)**

| **Items** | **Strongly Disagree** | **Disagree** | **Uncertain** | **Agree** | **Strongly Agree** | **Total Responses** |
| --- | --- | --- | --- | --- | --- | --- |
| 1. Chemotherapy protocols should be improved | 500 (13.2%) | 800 (21.2%) | 679 (18.0%) | 1200 (31.8%) | 600 (15.9%) | 3779 |
| 2. I have easy access to guidelines I need on managing chemotherapy resistance | 400 (10.6%) | 700 (18.5%) | 600 (15.9%) | 1300 (34.4%) | 779 (20.6%) | 3779 |
| 3. I have easy access to the materials I need to give advice on prudent chemotherapy use | 350 (9.3%) | 750 (19.8%) | 800 (21.2%) | 1200 (31.8%) | 679 (18.0%) | 3779 |
| 4. In the last 12 months, I received sufficient information about chemotherapy resistance | 200 (5.3%) | 600 (15.9%) | 600 (15.9%) | 1500 (39.7%) | 879 (23.3%) | 3779 |
| 5. On the basis of information I receive, I change my practice on prescribing/administering | 450 (11.9%) | 700 (18.5%) | 679 (18.0%) | 1200 (31.8%) | 750 (19.8%) | 3779 |
| 6. My country has a national action plan on chemotherapy resistance | 300 (7.9%) | 600 (15.9%) | 800 (21.2%) | 1300 (34.4%) | 779 (20.6%) | 3779 |
| 7. Chemotherapy resistance is very common in my practical settings | 250 (6.6%) | 550 (14.6%) | 900 (23.8%) | 1200 (31.8%) | 879 (23.3%) | 3779 |
| 8. I usually consult my colleagues about any case of chemotherapy resistance | 200 (5.3%) | 500 (13.2%) | 900 (23.8%) | 1400 (37.0%) | 779 (20.6%) | 3779 |
| 9. When there is chemotherapy resistance, alternative treatments should be used | 180 (4.8%) | 450 (11.9%) | 880 (23.3%) | 1450 (38.4%) | 819 (21.7%) | 3779 |
| 10. The sharing of information with patients on self-monitoring could be improved | 300 (7.9%) | 500 (13.2%) | 700 (18.5%) | 1200 (31.8%) | 1079 (28.6%) | 3779 |
| 11. I treat a patient with chemotherapy resistance similarly to those who do not have it | 250 (6.6%) | 470 (12.4%) | 850 (22.5%) | 1200 (31.8%) | 959 (25.4%) | 3779 |
| 12. Incidence of chemotherapy resistance is more common in specific types of cancers | 270 (7.1%) | 460 (12.2%) | 870 (23.0%) | 1100 (29.1%) | 1079 (28.6%) | 3779 |
| 13. Cross-resistance in chemotherapy can be detected and rectified in patients | 320 (8.5%) | 490 (13.0%) | 840 (22.2%) | 1050 (27.8%) | 1079 (28.6%) | 3779 |
| 14. Chemotherapy should be stopped immediately when it shows no signs of efficacy | 180 (4.8%) | 450 (11.9%) | 750 (19.8%) | 1300 (34.4%) | 1099 (29.1%) | 3779 |
| 15. Physicians, nurses, and healthcare personnel should be trained in detecting resistance | 220 (5.8%) | 500 (13.2%) | 880 (23.3%) | 1150 (30.4%) | 1029 (27.2%) | 3779 |
| 16. Oncologists should clearly explain consequences of chemotherapy resistance | 210 (5.6%) | 450 (11.9%) | 830 (22.0%) | 1300 (34.4%) | 989 (26.2%) | 3779 |
| 17. Therapeutic privilege among oncologists should be revised | 180 (4.8%) | 460 (12.2%) | 860 (22.8%) | 1200 (31.8%) | 1079 (28.6%) | 3779 |
